# Transfer of mode switching performance: from training to upper-limb prosthesis use

**DOI:** 10.1186/s12984-021-00878-4

**Published:** 2021-05-22

**Authors:** Anniek Heerschop, Corry K. van der Sluis, Raoul M. Bongers

**Affiliations:** 1 Department of Human Movement Sciences, University Medical Center Groningen, University of Groningen, Groningen, The Netherlands; 2 Department of Rehabilitation Medicine, University Medical Center Groningen, University of Groningen, Groningen, The Netherlands

**Keywords:** Assistive technology, Electromyography, Motor learning, Myoelectric control, Switching, Perception–action, Prosthesis, Transfer

## Abstract

**Background:**

Current myoelectric prostheses are multi-articulated and offer multiple modes. Switching between modes is often done through pre-defined myosignals, so-called triggers, of which the training hardly is studied. We evaluated if switching skills trained without using a prosthesis transfer to actual prosthesis use and whether the available feedback during training influences this transfer. Furthermore we examined which clinically relevant performance measures and which myosignal features were adapted during training.

**Methods:**

Two experimental groups and one control group participated in a five day pre-test—post-test design study. Both experimental groups used their myosignals to perform a task. One group performed a serious game without seeing their myosignals, the second group was presented their myosignal on a screen. The control group played the serious game using the touchpad of the laptop. Each training session lasted 15 min. The pre- and post-test were identical for all groups and consisted of performing a task with an actual prosthesis, where switches had to be produced to change grip mode to relocate clothespins. Both clinically relevant performance measures and myosignal features were analysed.

**Results:**

10 participants trained using the serious game, 10 participants trained with the visual myosignal and 8 the control task. All participants were unimpaired. Both experimental groups showed significant transfer of skill from training to prosthesis use, the control group did not. The degree of transfer did not differ between the two training groups. Clinically relevant measure ‘accuracy’ and feature of the myosignals ‘variation in phasing’ changed during training.

**Conclusions:**

Training switching skills appeared to be successful. The skills trained in the game transferred to performance in a functional task. Learning switching skills is independent of the type of feedback used during training. Outcome measures hardly changed during training and further research is needed to explain this. It should be noted that five training sessions did not result in a level of performance needed for actual prosthesis use.

*Trial registration* The study was approved by the local ethics committee (ECB 2014.02.28_1) and was included in the Dutch trial registry (NTR5876).

## Background

In current rehabilitation practice most individuals with a congenital reduction deficiency or an acquired upper limb amputation are fitted with a prosthesis. A substantial part of these upper-limb prostheses is controlled with myosignals recorded at specific muscles using surface electromyography (sEMG), hence, they are called myoelectric prostheses. Newer generations of such myoelectric prostheses are multi-articulating, i.e. the thumb and fingers have multiple joints enabling to perform pre-programmed modes (i.e., grip types and wrist motions). For some hands, up to 24 different grips are provided, such as opening and closing the hand, pinch grip, key grip, index pointing [1]. Although these modes increase the action opportunities of a user, they also present the user with a new challenge. Alternating or switching between more than two modes needs to be controlled while only a fixed and limited number of myosignals is available. The challenge for the user is that the number of myosignals is usually (much) smaller than the number of available modes. In recent literature three main solutions to this problem have been suggested; conventional control, pattern-recognition control, and simultaneous and proportional mapping [2].

In this paper we will focus on the conventional control strategy since this solution is currently used by the vast majority of multi-articulated hand prosthesis users. In this control strategy two myosignals, commonly derived from contractions of wrist extensors and flexors, are used to control the hand. Within the conventional control strategy the prosthesis user has to produce sequential-proportional signals [2, 3], which means that the hand first has to be switched to the appropriate mode after which this mode can be controlled proportionally. In order to grasp a glass for example, the hand first has to be switched to a power grip after which the hand can be proportionally opened and closed to pick-up the glass. Here proportional control indicates that the opening and closing speeds of the hand are proportionally scaled to the amplitude of the myosignals. Switching to the appropriate mode is achieved by generating predefined signals or ‘triggers’, which are distinctive sEMG signals from either the flexors or the extensors, or from both muscle groups simultaneously. Triggers that are often used in clinical practice are co-contractions; a very brief but powerful simultaneous contraction of the flexors and extensors, and double pulses; two brief contractions of one of the muscle groups [4].

We will focus on the mode switching aspect of sequential-proportional control since previous studies primarily focused on proportional control [5–11]. Where the effects and importance of training proportional control have been demonstrated, less is known about the effects of training mode switching. Note that training mode switching is relevant since from clinical experience we know that producing a co-contraction that meets the demands of the algorithm detecting a trigger is quite abstract and therefore a challenge for a lot of users. Previously, we have shown that roughly 50% of a group of able-bodied participants significantly improved their switching performance after training a short timeframe of 10 min [12]. However, it is unknown how performance changes over training programs of longer duration including more training sessions. Note that such a longer training more closely resembles actual rehabilitation practice. One of the challenges in applying a longer training duration is to keep patients motivated, which is not only relevant for the research setting but also for the rehabilitation practice. A promising way to keep users motivated for prolonged training might be exploiting serious games; games that are designed to entertain the trainee while at the same time teaching the trainee a new skill [7, 8, 11, 13–16]. Serious games might particularly be relevant for training prosthesis use since they can be used in the pre-prosthetic phase, when the actual prosthesis is not manufactured yet. Furthermore, different levels of difficulty can be adjusted to the user’s skill level and individualized feedback can be delivered to enhance effects of training. A first step in establishing how serious games can be used in training switching control, is to determine what kind of feedback is required for learning to switch. Therefore, in the current study we compared two training programs that varied in the feedback. One program entailed training with a serious game in which the only feedback was whether the trigger was correct or not, whereas in the other training program the actual myosignals were shown to the user. The serious game resembled the feedback during actual prosthesis use, whereas presenting the actual myosignals mimicks what is often done in rehabilitation training. Note that both training programs do not use a prosthesis, therefore it needs to be established that the skills obtained during training transfer to actual prosthesis use. Importantly, transfer has not been the focus of other studies assessing switch control and how it can be trained with a serious game [17–20].

The aims of our study were: (1) to assess whether mode switching control trained without a prosthesis transfers to mode switching control in actual prosthesis use and (2) to establish whether different types of training (i.e., manipulation of feedback) had a different effect on this transfer. (3) The third aim was to examine which clinical and myoelectric features of switching were adapted during training. We exploited two different training programs: a training program using a game with no feedback about the myosignal and a training program in which the trainee produced myosignals on a computer screen while receiving feedback on the quality of these signals. Results were compared to a control group. Elements of the employed serious game have already proven to be useful in transferring skills to actual prosthesis use after training proportional control [7, 8].

## Methods

### Participants

#### Sample size calculation

Data of the outcome measure ‘switching accuracy’ [12] was used to calculate the needed sample size per group. Switching accuracy was chosen since this outcome measure is clinically relevant for the prosthetic user. After a 10 min training period the mean (STD) of the switching accuracy was 26.60 (15.52).

Two sample size calculations were performed. The first calculation was performed to determine the sample size needed to detect differences between the pre- and post-test within groups. As a discriminant, we took a desired increase of 25% between pre-test and post-test. The second calculation was performed to determine the sample size needed to detect differences between the training groups. Two groups were considered statistically different if they had a 25% difference in accuracy after training. Using G*Power [21], aiming for a power of 0.95 and an α of 0.05, the needed sample size per group was respectively 6 and 10. We therefore aimed to include 10 participants per experimental group.

Twenty-eight participants were randomly assigned to the game group (GG) 10 participants (mean age 21.3 (3.2), 2 male, 8 female), the EMG group (EG) 10 participants (mean age 21.6 (1.9), 1 male, 9 female) or the control group (CG) 8 participants (mean age 21.1 (2.9), 2 male, 6 female) using a randomization program in Matlab. The CG was added to control for changes in performance that were not the direct result of the intervention/training.

All participants were able-bodied individuals, mostly students, of 18 years or older, who were right handed, had normal or corrected to normal vision, were free of any (history of) disorders of the arms and upper body and had no prior experience in using a myoelectric device. The experiment was performed at the Department of Human Movement Sciences (University of Groningen, University Medical Center Groningen, Groningen, The Netherlands). Written informed consent was obtained from all participants prior to the start of the experiment.

### Design

A pre-test, post-test design was applied with a 5 day training period in between the tests. For all groups the experiment was conducted on five consecutive days. All participants followed a training session of 15 min per day. The pre-test was conducted on the first day before the start of the first training and the post-test on the fifth day after finishing the last training.

Both experimental groups and the control group performed a different task during the training period between the pre- and post-test: (1) the GG played a serious game using the myosignals of muscles in the forearm to control a virtual grabber, (2) the EG trained producing triggers with their myosignals while looking at their myosignal patterns on a computer screen and (3) the control group (CG) played the same game as the GG, though they used the touchpad of the laptop to control the game. As such, the CG did not use myosignals to perform the task.

### Materials

#### Pre-and post-test

Participants used a myoelectric upper-extremity prosthesis simulator (see also [5, 22, 23]) to execute the adapted version of the clothespin test [24]. The myoelectric prosthesis simulator consisted of a shell socket in which the participant’s right hand was placed. Two sEMG electrodes (13E200 electrodes, MyoBock, Otto Bock Healthcare products, Austria) were positioned in the shell socket. The location of these electrodes was adjustable, so that they could be placed on top of the most prominent muscle bellies of the flexors and extensors of the wrist. The socket was connected to a splint, that was adjustable in length, in order to precisely determine the position of the socket and electrodes in relation to the hand. The used hand, an i-Limb Revolution Ultra (Touch Bionics), was placed at the end of the socket using a standard connector.

#### Serious game task

The GG participants played a myogame; a game controlled using the EMG signals derived from the muscles of the right forearm. This myogame was controlled using two electrodes (similar to those in the simulator) placed on the most prominent muscle bellies of the flexors and extensors of the wrist, attached by using an elastic sweatband. Marking electrode positions with a waterproof marker ensured identical electrode positions for pre-test, post-test and training sessions.

**Fig. 1:**
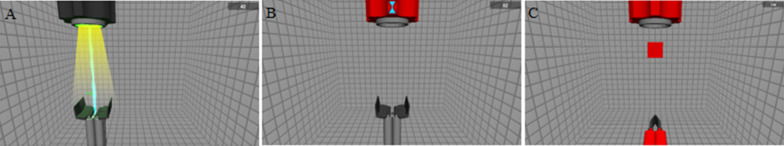
Serious game. The serious game consisted of three parts; **A** following a light beam with the platform to the left or to the right, **B** producing a mode switch within the time given as indicated by the hourglass (visible at the cannon in the top). A successful switch changed the platform into a grabber, and **C** catching a falling object using the grabber at the bottom

The electrodes were connected to a MyRio (National Instruments). LabView software (National Instruments Corporation, USA) on the MyRio was used to digitally filter (band filter, cut-off frequency 10 Hz; low pass filter, cut-off frequency 35 Hz) and calibrate (between 0 and 1, see "Procedure") the signals. The scaled signal was fed from the MyRio to a laptop computer via the wireless User Datagram Protocol (UDP) at 200 Hz. After calibration, participants had to play a game consisting of three parts; (A) following a light beam by controlling a laterally moving platform, (B) switching from the platform shaped controller to a virtual grabber, and (C) catching falling objects with the virtual grabber (Fig. 1). These three phases together comprised one repetition of the GG task. One level of the game consisted of 12 repetitions of the A–B–C phases. Correctly following the platform, producing the correct switch and catching the falling object earned the participants points. The scores were shown in the top right corner of the screen.

In part A of the game, the speed of the platform was proportionally related to the amplitude of the myosignals produced by the participants. Contracting the extensors of the wrist would move the platform to the right and contracting the flexors of the wrist would move the platform to the left. At the start of the trial the light beam moved in a certain direction and at a certain speed. This direction and speed did not change within a trial. Over trials the direction and speed varied. These settings were randomly changed throughout the level and were not within the participants’ control. In part B a co-contraction (trigger) had to be produced in order to switch from platform to grabber. An hourglass indicated the 15 s time window the participants had to perform the trigger. In part C the opening and closing speed of the grabber were proportionally related to the amplitude of the myosignal. Here the sEMG signal of the extensors and flexors of the wrist were used to open and close the grabber, respectively. The objects that had to be caught were all randomly sized between a fixed minimum and maximum size, they fell straight down from a cannon at the top of the screen. In the game, participants received feedback (see “Procedure”) about the width of the opening of the grabber relative to the object size and the closing speed.

#### EMG group task

**Fig. 2:**
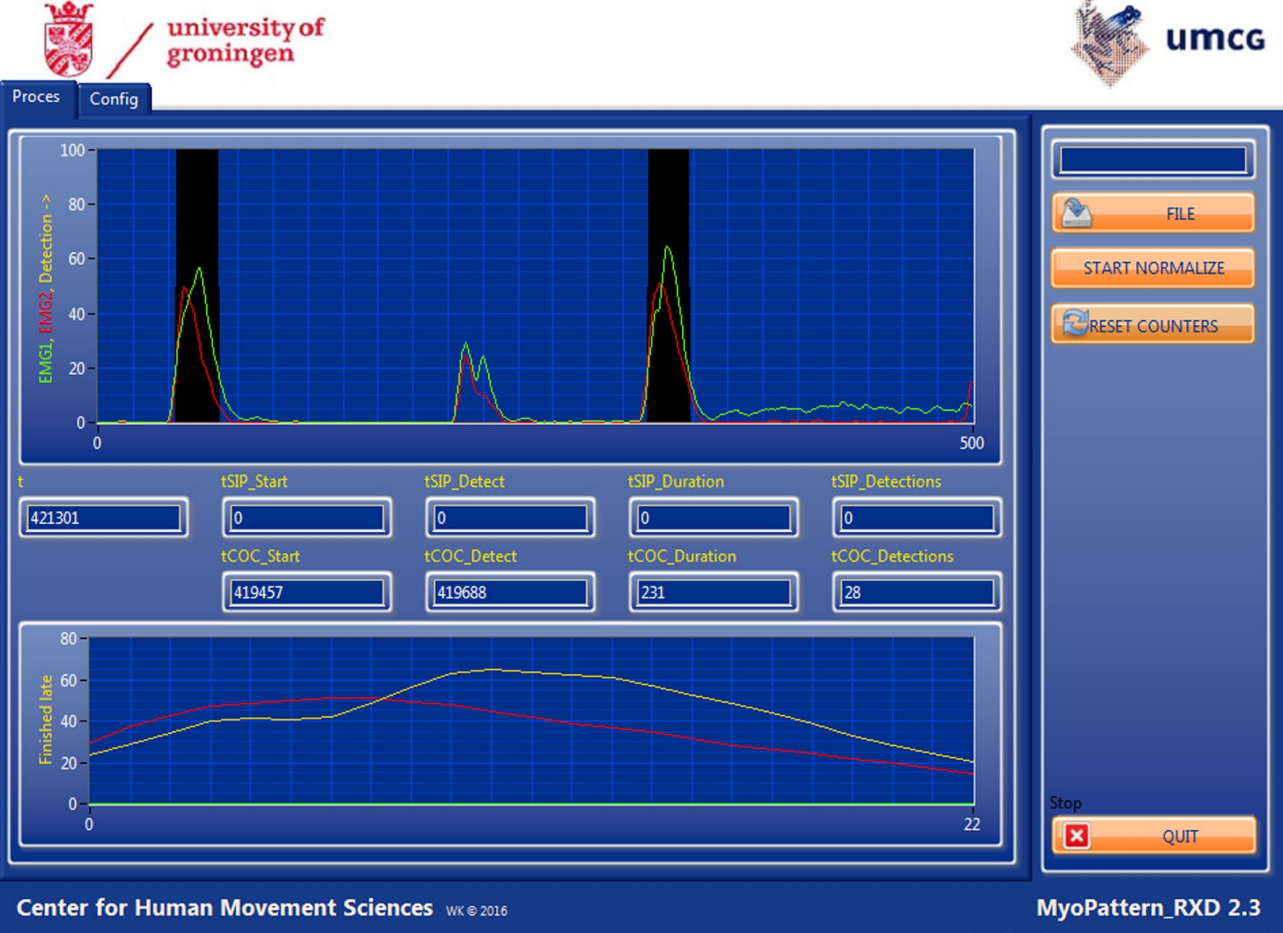
EMG feedback. The screen as seen by the EG group. In the left top half the myosignals were shown. Black squares indicated a correctly performed switch. At the left bottom half, the last correctly performed switch was visually enlarged

The EG participants saw their own myosignals on a laptop screen (Fig. 2, cf. [12]). Participants wore identical electrodes as in the GG with an identical configuration and filtering by the custom-made software on the MyRio. The scaled signal was fed to a laptop computer via a wired UDP protocol.

#### Control group task

The CG participants played the same serious game as played by the GG. They used the touchpad of the laptop to play the game.

### Procedure

Both the pre- and post**-**test commenced with palpating the muscles of the lower arm to find the most prominent muscles bellies of the flexors and extensors of the wrist, after which the prosthesis simulator was fitted. Subsequently, it was tested whether the participants were able to open and close the hand with ease. If this was not the case the prosthesis was fitted again and electrodes were slightly re-located until smooth opening and closing were assured.

#### Pre-test

All participants executed the adapted version of the clothespin test using the prosthesis simulator [24]. Since we were mainly interested in switching behaviour, the original clothespin relocation test was adapted in order to force participants to switch between grip-types after grabbing the clothespins, instead of being able to perform the task using compensatory movements. At the start of the test, the prosthesis hand was set in the neutral mode, and was placed on a pressure sensor. After hearing a beep, participants were allowed to lift the hand and start moving the clothespins. After moving the pins, the participant had to place the hand back on the pressure sensor.

**Fig. 3:**
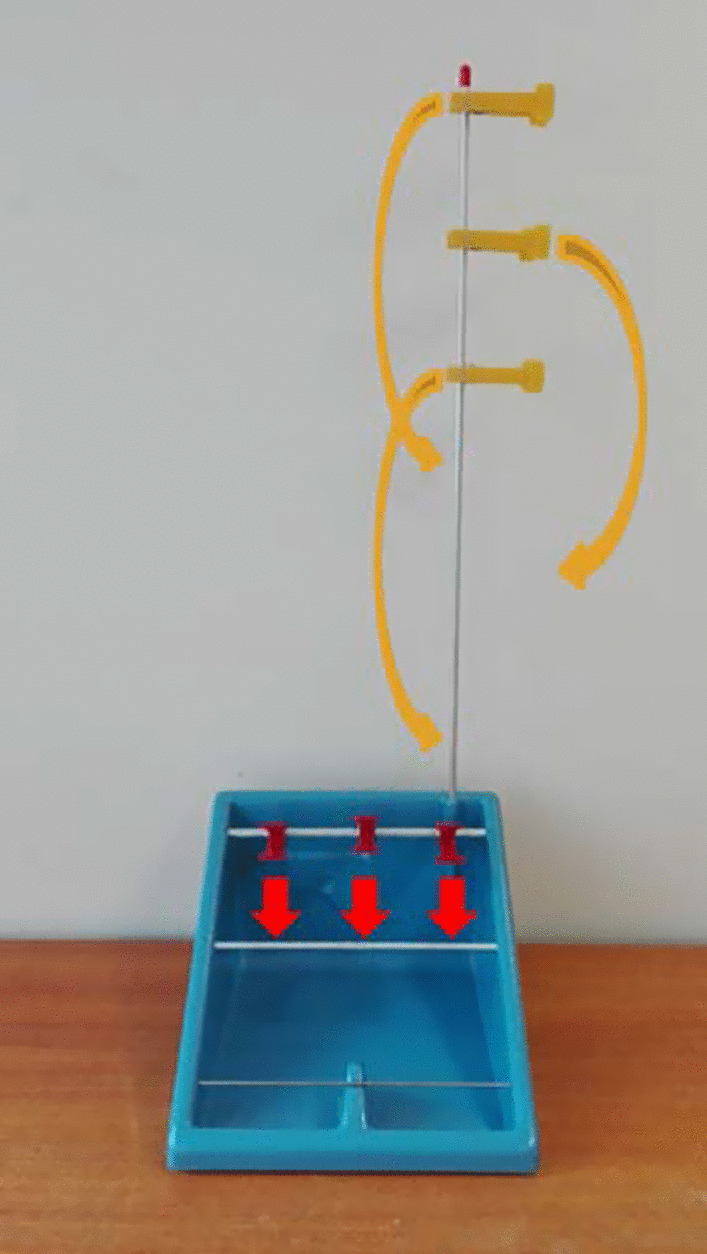
The adapted clothespin relocation task. The yellow, vertically placed clothespins had to be moved down using a fine pinch grip (see yellow arrows). The red, horizontally placed pins had to be moved to the lower bar using the key-grip (see red arrows)

During these tests participants were instructed to replace a total of six clothespins, three horizontally and three vertically placed ones, in alternating order. To pick up the horizontally placed pins a co-contraction had to be made to switch to the key-grip mode. To be able to pick up the vertically placed pins a double pulse had to be made to position the hand in the fine pinch grip mode (Fig. 3). The data on switching through double pulses is not presented in the current study. For all six triggers the participants had a time-frame of two minutes per trigger. If no trigger was made successfully within this window, a ‘hold open’ signal (3 s contraction of the extensor) had to be made to return to the neutral mode. When (by accident) a wrong trigger was produced the hand also had to be returned to the neutral mode. These accidental switches could occur when participants tried to stabilise the prosthesis. Verbal confirmation was asked to verify whether such a switch was made unintentionally. The six relocations of the clothespins were seen as six repetitions. Since we only present the data of the co-contractions in the current study, the 3 repetitions of the horizontally placed pins were analysed for both the pre and the post-test.

#### Training program GG

The GG training sessions commenced with the placement of the electrodes. To calibrate the sEMG signals, participants were asked to flex and extend their wrist slowly until they reached their maximum force. The maximum amplitude of the myosignal for both signals was set at 1, normalizing the signal. Participants were then instructed to follow the light beam as closely as possible with the platform being the avatar (see Fig. 1, part A) 1. If they did this correctly, a blue thunderstruck would appear and points would be awarded. In part B they were instructed to perform a co-contraction. If the switch was performed correctly, the plateau would reshape into a grabber for part C. If no correct switch was performed the screen would show part C but no action could be performed meaning the participant had to watch part C passively. If a correct switch was performed in part B, participants were instructed in part C to catch the object that fell from the top of the screen while avoiding crushing the objects by closing the grabber at the appropriate time and at the appropriate speed. This part of the game was similar to the one used by van Dijk et al. [7, 8]. The falling objects had different sizes and they could contain zero, one or two cracks resembling the three levels of breakability (unbreakable, moderately breakable or easy breakable). Furthermore, feedback was given on the aperture of the grabber. If this aperture exceeded 1.5 times the diameter of the object, the grabber would visually start to vibrate and give off sparks. Furthermore, opening the grabber 1.7 times the diameter of the object would cause it to close rapidly, making it impossible to catch the object. Participants were therefore also instructed to scale their hand opening and hand closing to the objects. Steps A-B-C were seen as one repetition. One level of the game consisted of 12 repetitions. Multiple levels, alternated with short breaks, were performed during the 15 min of training.

#### Training program EG

The EG sessions started in a similar way as the GG sessions. After calibration, participants were informed that they would see their sEMG signals on the screen in front of them. They were instructed to repeat the following: (A) producing maximum force with the flexor where the speed to build up that force could randomly vary between ‘slow’, ‘preferred speed’ and ‘fast’, (B) producing maximum force in the extensor muscles where the speed to build up that force was similar to the options in A, and (C) producing a co-contraction, where the requested switch had to be performed within 15 s. Subsequently participants started over with step A. The participants received feedback in the form of a black rectangle outlining the correct trigger if they had performed the co-contraction correctly (Fig. 2). Steps A–B–C were seen as one repetition and were repeated 12 times in one level. Multiple levels, alternated with short breaks, were performed during the 15 min of training.

#### Training program of control group

In the CG sessions the instructions given to the participants were identical to those received by participants in GG, besides that they were instructed to play the game with the mousepad and keyboard. The mousepad could be used to move the platform and to control the opening and closing of the hand. The number 1 on the keyboard could be used to perform a switch. No electrodes were fitted to the participants of this group. As for the GG, steps A–B–C were seen as one repetition. One level of the game consisted of 12 repetitions. Multiple levels, alternated with short breaks, were performed during the 15 min of training.

### Data handling and outcome measures

The data collected during pre- and post-test and during the training sessions were split into the repetitions as mentioned in the procedure. Within each repetition the relevant switching block was selected. This block started at the moment a switch had to be made and lasted until a switch was made (successful block) or the time window for that switch attempt came to an end (unsuccessful block). For the pre- and post-test these blocks had a duration of maximally 120 s, for the training these blocks had a maximum duration of 15 s. Hence, the successful blocks always lasted shorter than these maximum durations whereas the unsuccessful blocks always had these maximum durations. The outcome measures described below were calculated within these blocks and averaged per test or training session. To reduce effects of noise in the sEMG, detection of attempts to switch was based on an amplitude threshold of 20% of the maximum myosignal, which is similar to the threshold in the detection algorithm, and only attempts further than 0.5 s apart from each other were analysed. An attempt was only valid if at least one of the two sEMG signals exceeded a threshold of 20%. The reason for also including attempts where only one myosignal exceeded the threshold was that one of the difficulties of performing a co-contraction is the simultaneous contraction of both muscles. This often results in attempts consisting of one higher (above 20% of the threshold) and one lower (below 20% of the threshold) signal.

**Fig. 4:**
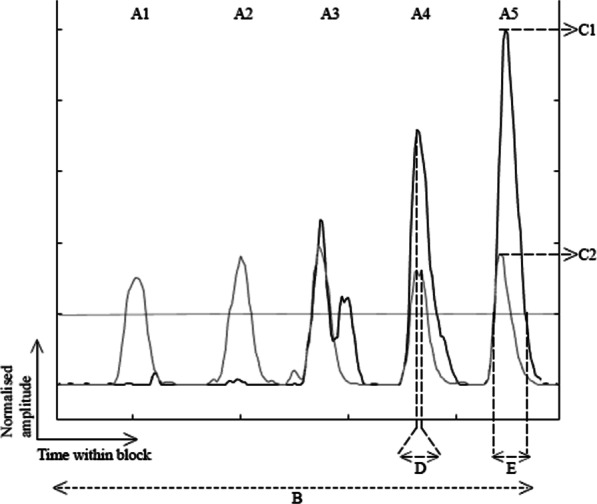
Representation of the outcome measures within one block. The grey and black lines show the myosignals from the wrist extensor and flexor during one training block. The horizontal line represents the threshold of 20% above which attempts were counted. The letters and symbols indicate the outcome measures (see Table 1): A1–A5: Attempts, B: Time needed to Switch, C1 & C2: Amplitude, D: Phasing, E: Width. Attempts A1 and A2 were unsuccessful since only one myosignal reached the amplitude threshold. The width of attempt A3 was too large to make a successful switch. In attempt A4 the phasing was too high. Attempt A5 was correct. The Accuracy of switching in this block was 1/5*100 = 20%

Six outcome measures were used to analyse the data. All outcome measures were calculated using customized Matlab (2019b, The Mathworks Inc., USA) scripts. An outcome measure was categorised as a clinically relevant performance measure (Table 1, three upper rows, and Fig. 4) or as a myosignal feature (Table 1, three bottom rows, and Fig. 4). The myosignal features were either derived from or identical to the parameters described by Tabor et al. [25].

**Table 1:** Definitions of the outcome measures

Outcome measure	Category	Representation in Fig. 4	Calculated for	Description
Attempts	Clinically relevant performance measures	A1–A5	Pre- and post-test, training sessions	Successful attempts, attempts resulting in a correct switch
				Unsuccessful attempts, attempts not resulting in a switch
				Number of attempts, number of attempts within a block. This measure was calculated for both successful and unsuccessful blocks
Time needed to switch	Clinically relevant performance measures	B	Pre- and post-test	The time needed to perform a correct switch. Calculated from the start of block till the moment a correct switch was detected. A shorter time needed to switch indicated better performance
Accuracy of switching	Clinically relevant performance measures	Computed from A1–A5	Pre- and post-test	Percentage of the attempts that resulted in a correct switch, calculated per block. The higher the accuracy of switching, the better the performance
Amplitude	Myosignal features	C1, C2	Training sessions	The height of the sEMG signal during a switch attempt. This measure was calculated for both sEMG signals separately and was averaged across the two (Amplitude = (C1 + C2) / 2)
Phasing	Myosignal features	D	Training sessions	The time difference between the peak amplitudes of both sEMG signals. Since ideally the peaks occur simultaneously, a smaller Phasing represents better performance
Width	Myosignal features	E	Training sessions	The duration between the start and end of a trigger command. The duration needed to remain below a 300 ms threshold in order for the algorithm to recognize it as a trigger. A shorter trigger duration reduces the likelihood for the algorithm to interpret the signal as an opening or closing command of the hand and thus reduces the chances of unintentional movement of the hand. Width was calculated based on the time between the myosignal that first reached the amplitude threshold (0.3) and the myosignal that last fell below this threshold. A smaller Width reflects better performance

### Pre- and post-test

For the pre-test and post-test all clinically relevant performance measures were calculated for successful blocks (i.e., blocks in which a switch was made and therefore had a duration shorter than 120 s). For unsuccessful blocks, the number of attempts during the time window of 120 s determined the ‘attempts’ value. Fixed values were used for the normality check and the comparison of groups within the pre- and post-test for the other two measures; ‘Time needed to switch’ equalized 120 as this was the maximum time available and ‘accuracy of switching’ equalized 0, since 0% of the attempts led to a successful switch. To analyse the differences between the pre- and post-test only the successful blocks were used.

### Training sessions

For the training sessions both the clinically relevant performance measures and the myosignal features were calculated (Table 1). The clinically relevant performance measures were calculated for the successful blocks and averaged per day. The myosignal features were calculated for every attempt separately. Attempts where then divided into successful and unsuccessful and for each measure the mean and the standard deviation were calculated per day.

### Statistical analysis

Using the Kolmogorov–Smirnov test it appeared that most outcome measures for the pre-test had non-normal distributions or were close to non-normal distribution. (Attempts needed for successful switch D(28) = 0.18, p = 0.021, Time till switch D(28) = 0.16, p = 0.067, Switching Accuracy D(28) = 0.31, p = 0.000). Therefor non-parametric tests were chosen for analyses.

To check for initial differences between groups, a non-parametric Kruskal–Wallis test for independent variables was applied to the pre-test data of the clinically relevant performance measures.

Subsequently transfer effects from training to prosthesis use (aim 1) were determined by analysing differences between pre- and post-test, using a non-parametric Friedman test for dependent variables (within group comparison). Differences in transfer effects induced by type of training programs (aim 2) were determined by using a non-parametric Kruskal–Wallis test for independent variables. This test was applied to the difference scores between the pre-test and post-test data (between group comparison).

To provide insight into changes in clinical and myosignal features of the sEMG during the 5-day training program (aim 3) the Pearson correlation coefficient (r) of the least square line of the outcomes per day across the 5 training days was computed for all outcome measures. For the outcome measures Accuracy and Amplitude a positive value of r indicated improvement. For the outcome measures Attempts, Time needed to switch, Phasing and Width a negative r value indicated improvement. Furthermore since we expected that over training the variability of the sEMG became smaller, a non-parametric Friedman test was used to see whether the interquartile ranges (IQRs) of the myosignal features changed over time. The IQR has been calculated per individual and those values are used in the Friedman test. This test thus analyses whether the median value of the within participant IQR differs. For all tests P-values smaller than 0.05 were considered to be statistically significant. If significant changes were found a post-hoc Wilcoxon signed-rank test with Bonferroni correction was performed to determine which training session(s) was/were significantly different from session 1.

## Results

### Pre-test data

The three groups did not differ on the pre-test for all clinically relevant performance measures indicating that the groups started at equal skill level: number of attempts χ^2^(2) = 0.63, p = 0.73, time needed to switch χ^2^(2) = 0.62, p = 0.73, accuracy of switching χ^2^(2) = 0.20, p = 0.90.

### Transfer of mode switching skill and the effect of training type

In order to determine if transfer occurred from training to prosthesis use we analysed the changes in performance from pre-test to post-test (within group comparison). Only the successful blocks were taken into account (Table 2) GG and the EG performed more successful blocks in the post-test compared to the pre-test. For the CG this was less evident.

**Table 2:** Number of successful blocks of replacing 3 horizontal clothespins during pre- and post-test

			Number of replaced clothespins within a block			
			3 Clothespins	2 Clothespins	1 Clothespin	0 Clothespins
GG	Pre-test	Number of successful blocks	5	3	–	2
	Post-test		8	–	1	1
EG	Pre-test		6	2	2	–
	Post-test		9	–	1	–
CG	Pre-test		5	2	–	1
	Post-test		4	3	–	1

*GG* game group, *EG*  EMG group, *CG*   control group

**Fig. 5:**
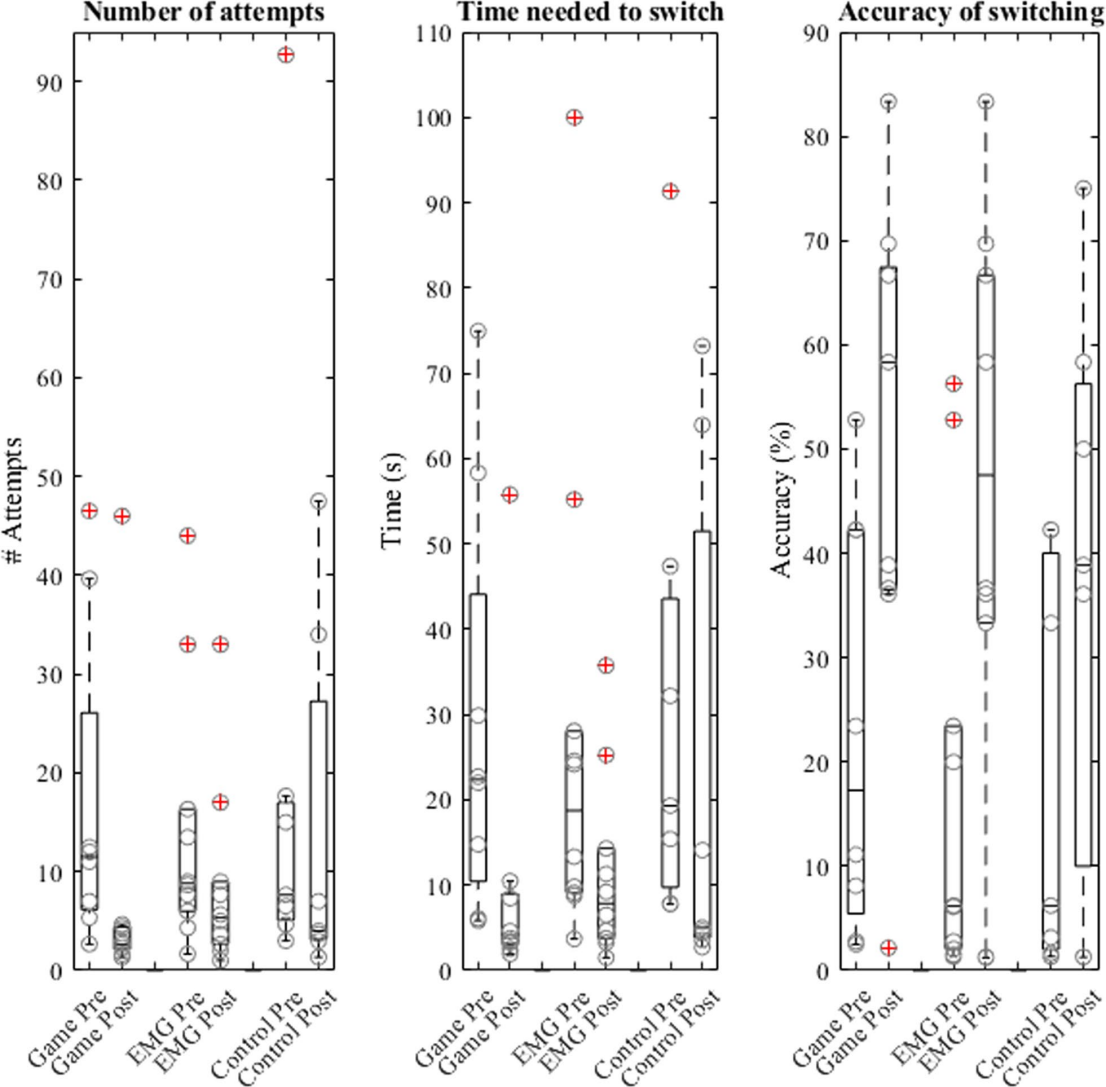
Boxplots for the clinically relevant performance measures. Left; Number of attempts needed in the successful blocks. Middle; Time needed to switch. Right; Accuracy of switching. In all images 6 boxplots can be seen. The first, second and third pair represent the pre- and post-test results of the Game Group, the EMG Group and the Control Group, respectively. Individual data points are shown as circles. Outliers are indicated with a red cross

GG and the EG showed an improvement from the pre-test to the post-test on all three clinically relevant performance measures (Table 3, Fig. 5), indicated by one significant effect (switching accuracy for GG) and marginally significant effects for all the other measures. The CG did not show significant differences between pre-test and post-test. Therefore, we focussed on the differences between the two experimental groups. A Kruskal–Wallis test on the difference between pre- and post-test scores showed no differences between the two experimental groups: number of attempts in successful blocks χ^2^(1) = 0.07, p = 0.79, time needed to switch χ^2^(1) = 0.01, p = 0.94, accuracy of switching χ^2^(1) = 0.01, p = 0.94. In short, we found differences between pre-test and post-test only for the two experimental groups indicating that transfer only occurred if sEMG signals were trained. However, the differences in feedback between the two experimental groups did not affect the transfer.

**Table 3:** Pre-test and post-test differences within groups

Outcome measure	Group	χ^2^- and p-values	Pre-test Median (IQR)	Post-test Median (IQR)
Number of attempts in successful blocks	GG	χ^2^(1) = 3.57, p = 0.06	11.50 (19.92)	2.67 (2.25)
	EG	χ^2^(1) = 3.60, p = 0.06	8.83 (10.33)	5.33 (6.33)
	CG	χ^2^(1) = 0.14, p = 0.71	7.67 (11.88)	4.00 (24.08)
Time needed to switch	GG	χ^2^(1) = 3.57, p = 0.06	22.37 (33.64)	3.77 (5.88)
	EG	χ^2^(1) = 3.60, p = 0.06	18.76 (18.98)	7.85 (10.55)
	CG	χ^2^(1) = 1.29, p = 0.26	19.32 (33.85)	5.00 (47.62)
Accuracy of switching	GG	χ^2^(1) = 7.00, p < 0.01*	17.28 (36.79)	58.33 (30.90)
	EG	χ^2^(1) = 3.60, p = 0.06	6.17 (21.38)	47.50 (33.33)
	CG	χ^2^(1) = 1.29, p = 0.257	6.22 (37.98)	38.89 (46.25)

Values with * indicate a significance level of p < 0.01

*IQR* interquartile range, *GG* game group, *EG* EMG group, *CG* control group

### Adaptation during training

For GG and EG we considered changes in both the clinically relevant performance measures and the myosignal features over the training sessions. This aim only concerns GG and EG since in CG the sEMG signals were not used for controlling the task.

#### A. Clinical relevant performance measures

**Fig. 6:**
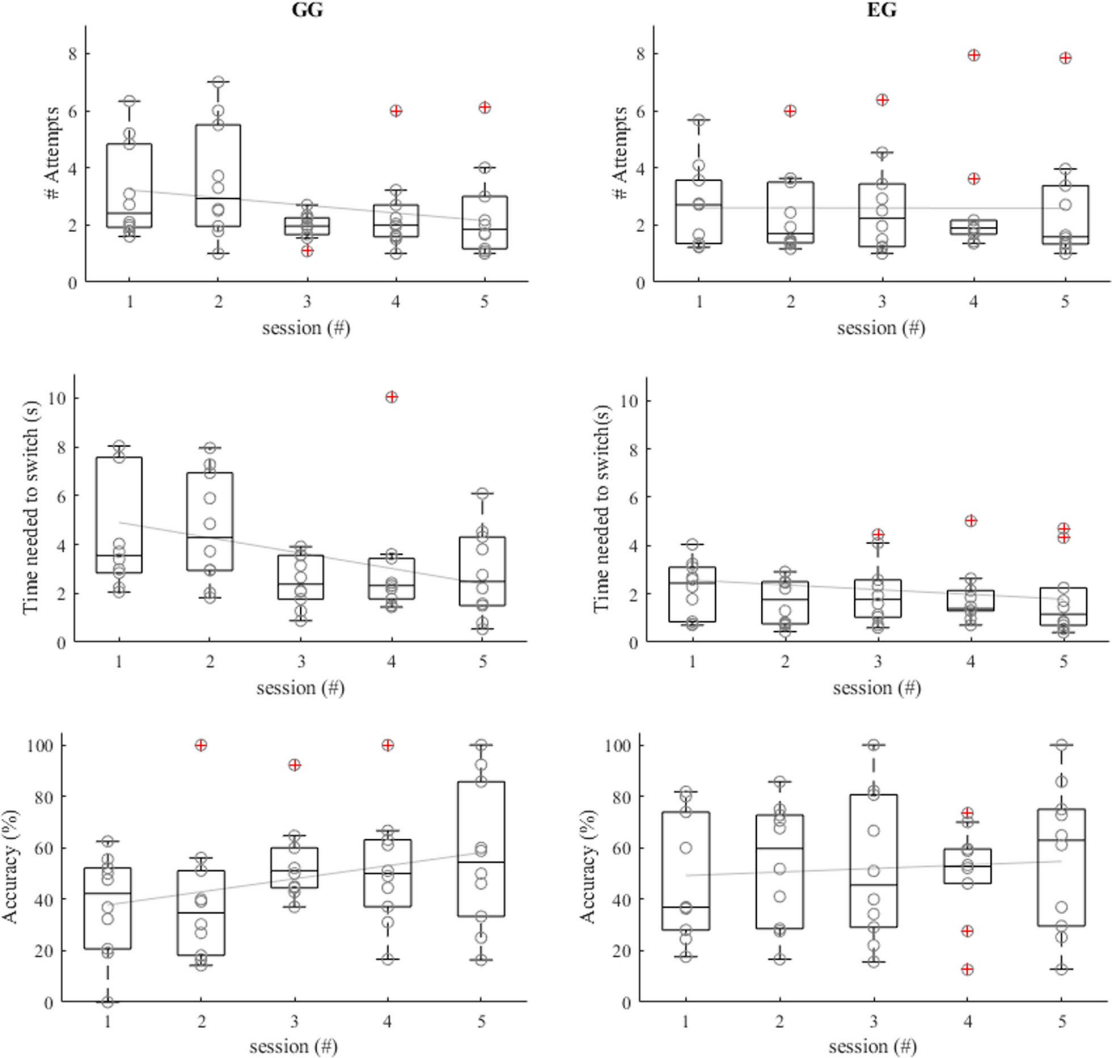
Boxplots of the clinically relevant performance measures during the training sessions for GG (left) and EG (right). Individual data points are shown as circles, these are the means of all successful blocks per training session. The least squares line is plotted in grey in the background. Outliers are indicated with a red cross

In GG, Time needed to switch decreased marginally significant and Accuracy of switching significantly increased over the 5 training days (r = − 0.83, p = 0.08 and r = 0.91, p = 0.03 respectively) (Fig. 6). Both outcomes measures reflected improvement of switching skills. No further significant changes were found.

#### B. Myosignal features

**Fig. 7:**
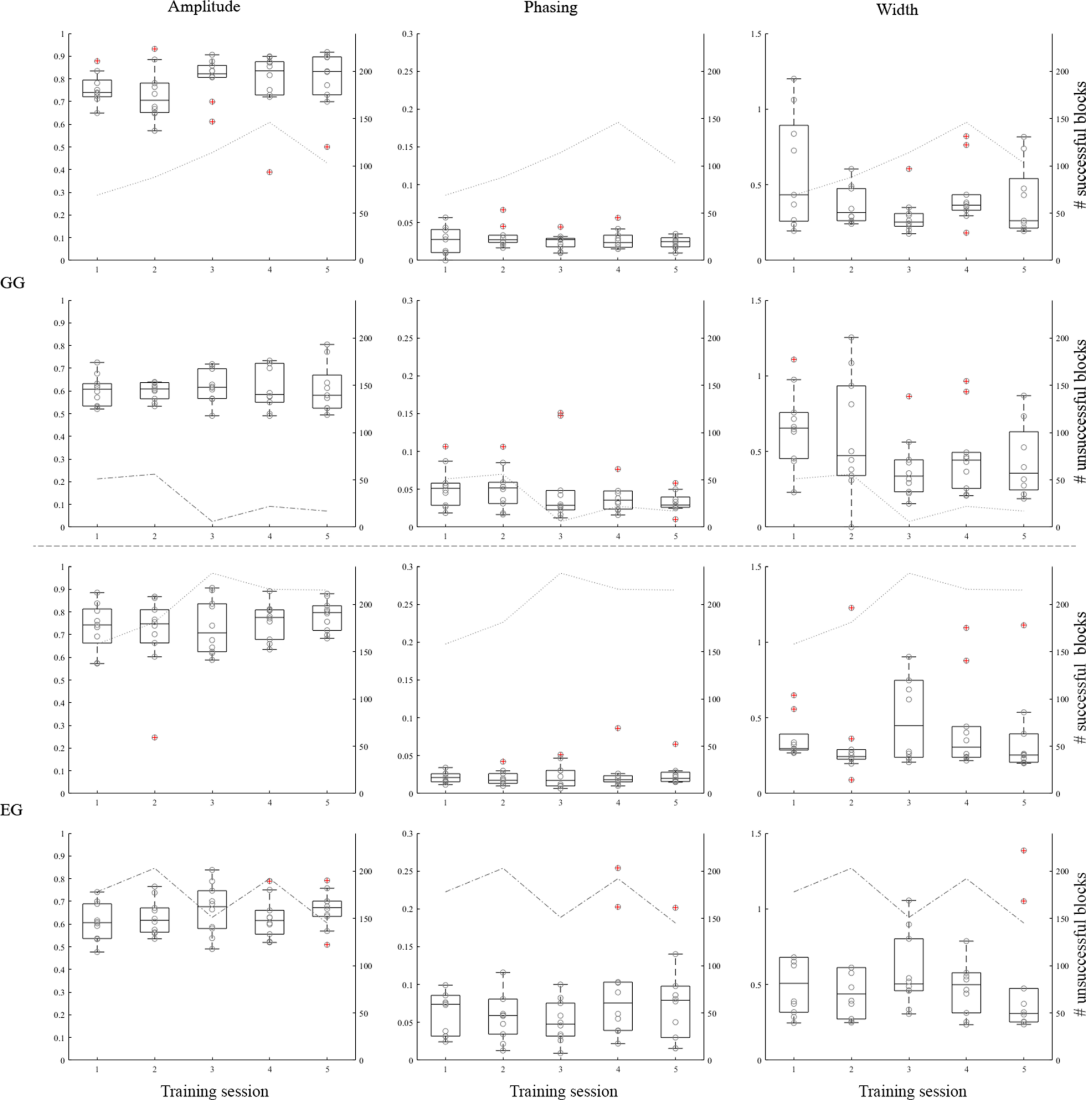
Boxplots of the myosignal features over training for GG (upper 6 panels) and EG (lower 6 panels). For both groups, the top three images show outcomes for the successful switches, the bottom three images show the outcomes for the unsuccessful switches. The left column shows the amplitude, the middle column the Phasing and the right column the Width. All images show the median and quartile ranges of the group for a specific outcome measure. Outliers are marked in red. The dotted grey line in row 1 and 3, and the dashed line in row 2 and 4 show the total number of successful or unsuccessful blocks for all participants within the two groups, respectively (scale on the right y-axis)

The variance within the measures was expected to decrease over time because participants would become more stable in producing the triggers over training. However, only the IQR of the Phasing in the successful blocks for the EG showed significant differences between the 5 sessions (χ^2^(2) = 12.326, p = 0.02). The post-hoc test showed that only the IQR of Phasing was significantly lower (i.e. better) during training session 3 compared to training session 1 (Z = − 2.380, p = 0.017). Note, this significance cannot be seen in Fig. 7. In this figure the group mean per day is represented to show progression over the training sessions. However in the calculation of the variance within the measures the IQR has been calculated per individual and those values were used in the Friedman test.

In addition, we observed some trends in the data after perusing the data. Since these trends were not the primary focus of this paper but were incorporated to provide more information for other researchers in the field, we briefly describe them here. For both GG and EG the total number of successful blocks (Fig. 7, rows 1 and 3) showed an increasing trend while the total number of unsuccessful blocks showed a decreasing trend (Fig. 7, rows 2 and 4), indicating that both groups seem to have improved over time. Note that deviations from this trend might have originated from some participants showing deviating patterns on the last day. Moreover, some differences between the successful and unsuccessful switches can be observed in Fig. 7. These differences are all in the direction as what was to be expected. The amplitude of the sEMG peaks seemed larger for successful than for unsuccessful switches (Fig. 7). Phasing and Width appeared to be smaller for successful switches than for unsuccessful switches. For Phasing the standard deviation appeared lower for the successful switches.

## Discussion

The focus of the current paper was on the requirements of training to produce triggers to switch the mode of a multi-articulated myoelectric prosthesis hand. Learning to produce these triggers has received scant attention in the literature (but see Tabor, Heerschop, Prahm), while this skill is relevant for numerous users to control their multi-articulated prosthetic hand in daily life. A first step in improving training is establishing the feedback required during training, hence we compared two groups that differed in the feedback about the myosignal when switching. Moreover, it is important that the skill in the training transfers to actual prosthesis use, hence this was examined separately. One of the training groups trained with a serious game in which only feedback about the correctness of the produced trigger was provided, resembling feedback during actual prosthesis use, whereas the training was playful. The other training group could see the produced trigger online on a screen and received feedback about required changes of the myosignal. We found that both training groups showed significant and marginally significant transfer from training to prosthesis use while the control group did not. The results demonstrated that specifics of the training did not affect transfer. Stated otherwise, training with a serious game gave no significantly different transfer effect than training while seeing the produced myosignal. Although we did not find differences between the groups in transfer, the two training groups showed differences during training. Where the gaming group significantly increased in accuracy of the switches over training the EMG group did not. And where the group receiving feedback about the myosignal improved in the variability of the phasing of the peaks in the myosignal during one of the training session the game group showed no significant changes in myosignal measures.

### Transfer of switching skill and the effect of training on that transfer

Our findings showed that training switching does transfer to actual prosthesis use, and that training is relevant to learn to produce accurate switches in a short time in a functional task. Importantly, the actual way in which the production of the switch is trained does not affect the transfer to actual prosthesis use, at least not when comparing our experimental manipulations (i.e. properties of the game and the presentation of the myosignals). Our findings imply that the relevance for training should not be underestimated, it also seems to be important for a task as abstract as producing a trigger.

The current study focussed on the feedback about the myosignal that should be presented during this training. We chose to assess two extreme versions of feedback; one group trained with a serious game where a user only received feedback about the outcome of the produced trigger, whereas the other group could see the produced myosignal and received feedback about what should be adjusted in the myosignals. We chose these two training groups because they both have clinical relevance: presenting the outcome of the produced trigger resembles actual prosthesis use, while the total EMG signal is often presented in clinical rehabilitation training. That these extremes do not differ in the effect of the training on the transfer to actual prosthesis use might imply that the actual feedback is not conclusive in the effect of training the trigger production. Therefore, we suggest that future studies aiming at improving training for trigger production should also focus on other aspects than feedback, such as the duration of the training, the optimal interval between trainings, and for serious game applications the game environment that provides highest engagement and motivation (cf. [17–20]).

We would like to emphasize that our study is different from most other studies on applications of serious gaming to train mode switching in prosthesis use because we also focused on the transfer to actual prosthesis use. In contrast, most other studies focused on motivation and engagement [17–20, 26] and/or improvement of the properties of the myosignal [12, 18, 26] during training. For example, Prahm et al. [18] investigated people with an upper limb amputation who were mostly new to prosthesis control and who played three games in a single session. The authors measured motivation and properties of the myosignal, such as accuracy of control. Properties of the myosignals were compared between pre-gaming and post-gaming using specific EMG tests. Their results showed improvements in overall EMG control, fine muscle activation and separate activation of the EMG electrodes. Although these findings are important and support our findings regarding the relevance of training, it is important to establish that training improves the use of different grip types in prosthesis use in functional tasks (cf. Van Dijk, [8]). Therefore, in the current study we set out to examine whether the training improved the use of different grip modes in the Clothespin task. We found that training indeed is relevant and the skills trained in the game indeed transfer to performance in a functional task.

### Changes during training

The fact that training switching did affect switching performance in prosthesis use raises the question as to which aspects of the trigger changed during training. During training we assessed clinical performance measures and features of the myosignal (Figs. 6 and 7, respectively). In the analyses of the data collected during training the clinical performance measures within the two training groups showed that for the GG the time needed to switch and the accuracy of switching improved while this was not the case for the EG. This is remarkable since, due to the set-up of the study, the GG spent less time training switching behaviour (see limitations) but nonetheless they improved on two measures that are clinically relevant for prosthesis users. One possible origin of this effect could be that playing the game was more motivating than looking at a screen with myosignals presented on it. This suggestion is substantiated by informal comments of the participants during practicing with the game. Participants remembered their own scores and informally expressed motivation to beat their own high scores. This suggests that gamification of training might lead to better training results.

With respect to the myosignal features we expected a decrease in variability of the myosignal features as this would reflect a more stable behaviour after training. Only one significant change in IQR was however found; variability in phasing in the EG was significantly lower during session 3 compared to day 1. Importantly, we used features of the myosignals that were similar to those that Tabor et al. [25] assessed. They found that only a few features of the trigger changed over training. We chose to analyse these features since these features were most distinctive for the detection of the trigger. Moreover, these features could be assessed on the actual triggers produced during training and potentially also during prosthesis use. Interestingly, Prahm et al. [18] measured other aspects of myosignal properties and found an effect over a short training. However, these measures required separate tests and would in a daily life situation require additional effort of users.

Our findings suggested that after a period of training no large changes in the myosignal features had taken place on group level. However, since we found an improvement on performance level between pre- and post-test, certain myosignal features (which may be undefined yet), must have changed. An observation within our study was the large difference between individuals, which might be important in establishing and devising new avenues for research on myosignal features during switching and their change over training. Of the many further steps yet to be taken to study myosignal features in order to better understand what is learned within the specific task of switching, future research should focus on differences in learning between individuals inspired by papers that describe the possibility that all individuals have their own learning strategy (cf. Golenia et al. [27] and Pacheco and Newell [28]). Following this approach would imply that analyses on myosignal features should not be performed on group means but on individuals’ data.

In our previous study [12], where switching was trained using two short 10 min sessions, we found statistically significant changes in myosignal features within this relatively short period of training. We assume the difference between our current and the previous study can be explained by the steep learning curve at the beginning of the training while this learning curve will become flatter after some practice. However even though the learning curve is flatter in later parts of the program, more subtle, smaller, but perhaps no less important changes are likely to occur in the later training sessions. As indicated in the previous paragraph, looking at individual data might give more insight in these changes within the flatter learning curve. The finding that not many improvements in the measures used to assess the myosignal were found, is remarkable given that the training transferred to functional prosthesis use. Users must have picked up on the perceptual information guiding the production of the trigger, however, from the current findings we could not derive what it is that is learned in producing the trigger, let alone the perceptual information guiding this behaviour. This idea that the perception–action coupling in switching of modes using a trigger is not straightforward is in line with the clinical reports that the task of switching is unnatural and unintuitive, which is one of the biggest challenges in the conventional control strategy. This was confirmed by Franzke et al. who recently showed that prosthesis wearers found the process of switching time consuming, unreliable, non-intuitive and mentally exhausting [29]. Kuiken et al. reported similar findings in several patients who perceived switching as inconvenient [4]. Our results in non-prosthesis wearers support these reports. Even though the time needed to switch improved over training, in general (at least) 4 s were needed to switch a prosthetic hand from one mode to another. For prosthesis users such a lengthy time needed to switch will not stimulate them to switch grips often. Instead, they persist using the current mode and make use of compensatory movements in trunk and/or shoulder to perform the desired activity in daily life. Furthermore, nearly all participants produced failures and needed at least three attempts before a successful switch could be made. In daily life prosthesis use this will cost much energy and patience, again most likely resulting in patients using compensation strategies instead of switching. It might be that a longer training duration may lead to better results. For some individuals this will be sufficient to meet their needs, for example if they plan on using two grip types and training enables them to be proficient in one switch command. However, depending on the wishes and the skills of the wearer, we feel that other avenues of prosthesis control of multi-articulated hands should also be explored. For example gesture control, as employed in the latest versions of the iLimb hands (Touch Bionics by Össür), or pattern recognition control [4, 30–32] might be more suitable for other individuals.

### Limitations

The GG performed less blocks during the training program than the EG group, due to the fact that the proportional parts of the GG task took longer than those in de EG task. GG might have performed better if duration times would have been equal.

The thresholds for the amplitude and the rise and fall times for the EMG as implemented in our software were based on online values provided by Touch Bionics by Össür. During the analysis we found that our requirements were stricter than the requirements that were implemented in the actual prosthesis hand. Since identical software was used by both training groups it did not affect the comparison between the groups, however the transfer effect might have been even stronger if identical requirements had been used.

In this study the participants trained with two electrodes covered by a sweatband. An actual prosthesis consisting of a socket and a hand will add weight and therefore pressure on electrodes and increase strain on the muscles. This will alter the interaction between the muscles and the electrodes and may provide different results. Furthermore, healthy participants were included instead of persons with an upper limb defect. The reasoning behind this choice was that we chose to first test our ideas in adults without an impairment to increase our knowledge on switching behaviour and myosignal changes. Having done that we can now refine our protocols and formulate more precise research questions before setting up a study including individuals from the rather small population of people with an arm amputation. The sEMG derived from a stump is different from sEMG derived from unaffected limbs [33]. We advise therefor to replicate the current study in prosthesis users to validate our results.

## Conclusion

The current findings demonstrated that training is important to improve switching the mode of a multi-articulated prosthesis hand using a EMG trigger. Comparing different types of training where participants trained with a serious game or with the myosignals presented on a screen did not affect the results. Both trainings transferred to actual prosthesis use and this deserves more attention in future studies. Only a few clinically relevant variables and properties of the myosignals changed during training which is remarkable. Although the training affected the switching performance, the time to produce a switch was still too long for the switching to be effective in daily life.

## Data Availability

The datasets used and/or analysed during the current study are available from the corresponding author on reasonable request.

## Abbreviations

CG: Control group
EG: EMG group
GG: Game group
sEMG: Surface electromyography
UDP: User datagram protocol
